# Synthesis of New RE^3+^ Doped Li_1+*x*_Ta_1−*x*_Ti*_x_*O_3_ (RE: Eu, Sm, Er, Tm, and Dy) Phosphors with Various Emission Colors

**DOI:** 10.3390/ma6072768

**Published:** 2013-07-11

**Authors:** Hiromi Nakano, Shiho Suehiro, Shohei Furuya, Hiroyuki Hayashi, Shinobu Fujihara

**Affiliations:** 1 Toyohashi University of Technology, Tempaku, Toyohashi 441-8580, Japan; E-Mails: s113517@edu.tut.ac.jp (S.S.); s123440@edu.tut.ac.jp (S.F.); 2 KRI, Inc., Chudoji Minami-machi, Shimogyo-ku, Kyoto 600-8813, Japan; E-Mail: h-hayasi@kri-inc.jp; 3 Keio University, Yokohama 223-8522, Japan; E-Mail: shinobu@applc.keio.ac.jp

**Keywords:** solid solution, host material, phosphor, structure, photoluminescence

## Abstract

New phosphors with various emission colors for RE^3+^ doped Li_1+*x*_Ta_1−*x*_Ti*_x_*O_3_ (LTT) (RE: Eu, Sm, Er, Tm, and Dy) were synthesized by electric furnace at 1423 K for 15 h. The microstructure of the host material and the photoluminescence (PL) property were determined and compared to those of RE^3+^ doped Li_1+*x*_Nb_1−*x*_Ti*_x_*O_3_ (LNT)_._ In the LTT phosphor, the highest PL intensity was achieved for the mixture composition Li_1.11_Ta_0.89_Ti_0.11_O_3_ with a LiTaO_3_ structure, although it has an M-phase superstructure. In the LTT host material, the effective activators were Eu^3+^ and Sm^3+^ ions, in contrast to the LNT host material. Here, we discuss the relationship between PL property and the host material’s structure.

## 1. Introduction

In the ternary Li_2_O-M_2_O_5_-TiO_2_ system, Li_1+*x*−*y*_M_1−*x*−3*y*_Ti*_x_*_+4*y*_O_3_ (M: Nb or Ta, 0.06 ≤ *x* ≤ 0.33, 0 ≤ *y* ≤ 0.17) forms with a superstructure, and this is known as the M-phase. Since the discovery of the M-phase for Li_1+*x*−*y*_Nb_1−*x*−3*y*_Ti*_x_*_+4*y*_O_3_ (LNT) by Castrejon* et al.* [1,2], such structures have been investigated [3,4,5,6]. The M-phase superstructure is formed by periodical insertion of an intergrowth layer in the matrix having a trigonal structure. The relationship between dielectric property and period of the intergrowth layer of the M-phase has been studied [7,8]. To apply this unique structure to the host material of a phosphor, new phosphors have been investigated based on LNT synthesized by a conventional electric furnace [9,10]. A homogeneous material was, however, synthesized at 1373 K for 24 h. We have succeeded in synthesizing an LNT solid solution having a superstructure by millimeter-wave heating for only 1 h above 1173 K [11]. The technique has also been applied to the synthesis of rare-earth doped LNT phosphor [12,13]. The photoluminescence (PL) intensity of LNT:Eu^3+^ at 625 nm was much higher than that of LiNbO_3_:Eu. [12] Rare-earth-doped LiTaO_3_ (RE^3+^: Pr^3+^ [14], Er^3+^ [15], Tb^3+^ [16] Eu^3+^ [17] and Tm^3+^ [18]) have also been reported by other groups. Recently, we have reported a new red phosphor based on the quaternary Li_1+*x*_ (Ta_1−*z*_Nb*_z_*)_1−*x*_Ti*_x_*O_3_ (LTNT, 0.05 ≤ *x* ≤ 0.25, 0 ≤ *z* ≤ 0.625 ) solid solution as a host material. The PL intensity of the LTNT:Eu^3+^ phosphor was found to be dependent on the concentration of Eu^3+^, Ti^4+^, Nb^5+^, and Ta^5+^ ions [19].

In this work, rare-earth doped Li_1+*x*_Ta_1−*x*_Ti*_x_*O_3_ (LTT) phosphors with various emission colors were synthesized by an electric furnace and compared to LNT phosphors for annealing condition, host material’s structure, and PL property.

## 2. Results and Discussion

**Figure 1:**
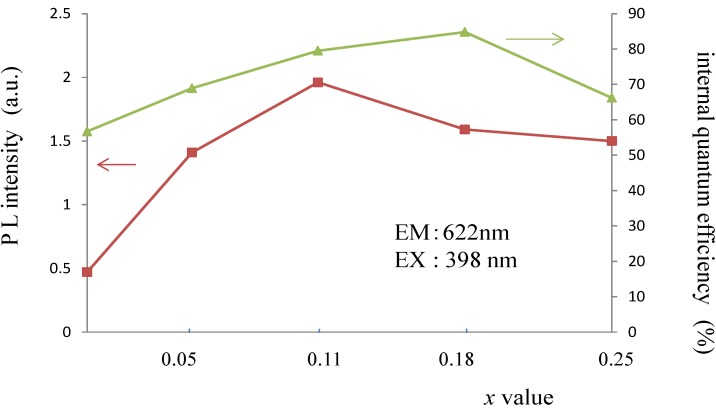
Relationships among PL intensity, internal quantum efficiency, and TiO_2_ content in Li_1+*x*_Ta_1−*x*_Ti*_x_*O_3_ host material.

As we reported previously for the Li_1+*x*−*y*_Nb_1−*x*−3*y*_Ti*_x_*_+4*y*_O_3_ (LNT, 0 ≤ *x* ≤ 0.25, *y* = 0) system [12], the PL intensity was clearly improved by the addition of TiO_2_, which could affect the coordination state of the Eu^3+^ ion and/or induce a structural distortion around this ion. The most important factor is thus expected to be the TiO_2_ content in the Li_1+*x*_Ta_1−*x*_Ti*_x_*O_3_ (LTT) solid solution. The optimal TiO_2_ concentration was then determined at the maximum emission peak upon excitation at around 399 nm. Red emission of the Eu^3+^-doped LTT was observed at an excitation wavelength of 399 nm due to the intraconfigurational ^7^F_0_-^5^L_6_ transition. The maximum emission peak at around 624 nm is associated with the hypersensitive electric-dipole ^5^D_0_-^7^F_2_ transition in the Eu^3+^ ion. Figure 1 shows the relationships among the PL intensity, internal quantum efficiency, and the TiO_2_ content in the ternary Li-Ta-Ti-O system upon varying the TiO_2_ content from* x* = 0 to 0.25 at a fixed Eu_2_O_3_ concentration of 2.5 wt %. The highest PL intensity is found for the composition of Li_1.11_Ta_0.89_Ti_0.11_O_3_ (*x* = 0.11, *y* = 0), where the chemical composition is based on the mixture ratio. The internal quantum efficiency revealed a high value of 84.8%, but the highest value appeared in Li_1.18_Ta_0.82_Ti_0.18_O_3_ with* x* = 0.18. The discrepancy in the *x* values that give the highest PL intensity and the highest quantum efficiency may come from the different measurement apparatus. Because the integrating sphere was used in the internal quantum efficiency measurement, the “*x* = 0.18” value is supposed to be more appropriate to discuss the relationship between the host crystal composition and the emission properties. The incorporation of Eu^3+^ ions in the crystal structure of LTT would cause the overlapping of orbitals with the adjacent anions, resulting in efficient red-light emission due to the hypersensitive ^5^D_0_-^7^F_2_ transition in Eu^3+^.

**Figure 2:**
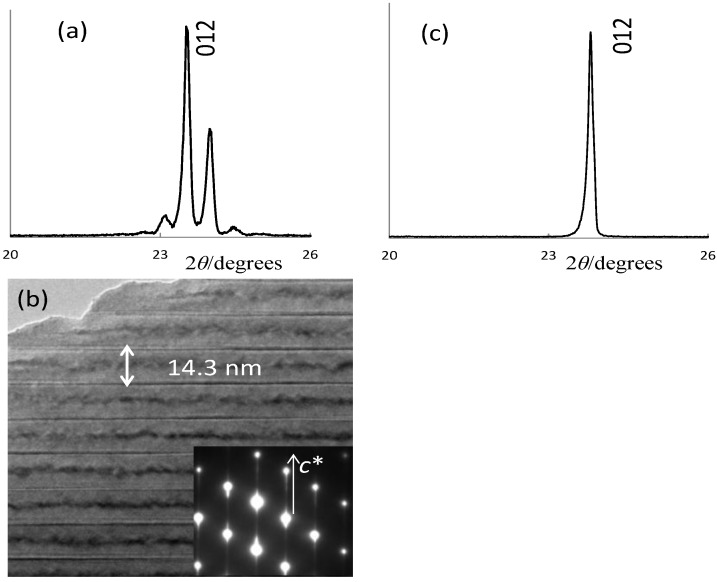
Structures of Li_1.11_M_0.89_Ti_0.11_O_3_ (M: Nb or Ta) host material: (**a**–**b**) LNT; and (**c**) LTT.

The LNT phosphor with composition of Li_1.11_Nb_0.89_Ti_0.11_O_3_: *x* = 0.11 was also found to have the highest PL intensity [9]. Here, the host material’s structure was compared between LNT and LTT. In Li_1.11_Nb_0.89_Ti_0.11_O_3_, the superstructure is formed by periodical insertion of the intergrowth layer with 14.3 nm spacing into a matrix with a trigonal structure (Figure 2b), and satellite reflections appeared around the (012) in the XRD pattern (Figure 2a). The superstructure was formed by doping of the Ti^4+^ ion, and the period was controlled by the Ti content, as described in our previous paper [11]. On the other hand, Li_1.11_Ta_0.89_Ti_0.11_O_3_ has no superstructure but only a basic structure of LiTaO_3_ (Figure 2c). The results revealed that the composition area of the M-phase with a superstructure is different between LNT and LTT. In our recent paper [20], the refined structure was compared between LiTaO_3_ and Li_1.11_Ta_0.89_Ti_0.11_O_3_ by the Rietveld method from powder X-ray diffraction data. The composition was determined as Li(Ta_0.89_Ti_0.11_)O_2.945_ from the final structure model, in which the Ti ion is located at the Ta site. By doping of the Ti^4+^ ion into LiTaO_3_, the <Li-O> distance of the (LiO_12_) polyhedron in the LiTaO_3_ structure (*R*3*c*) changed from 0.274 nm to 0.270 nm, and the [(Ta,Ti)O_6_] octahedra are all comparable with each other. As a result, the structure is stable without a superstructure.

**Figure 3:**
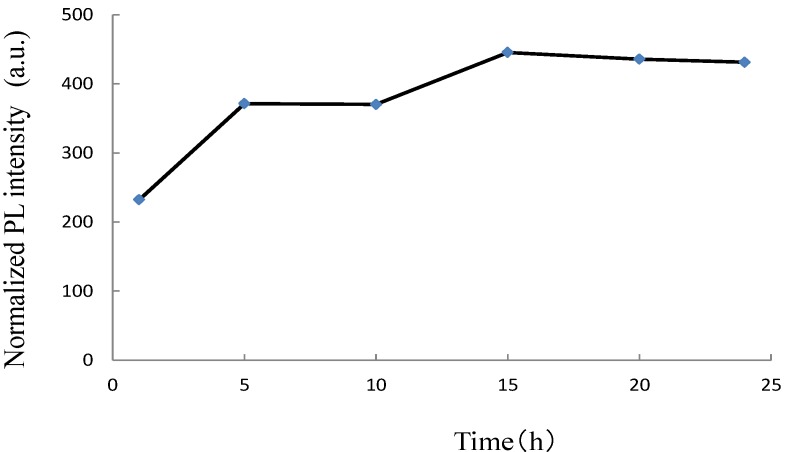
Relationship between PL intensity and annealing time in Li_1.11_Ta_0.89_Ti_0.11_O_3_:Eu^3+^ phosphor.

**Figure 4:**
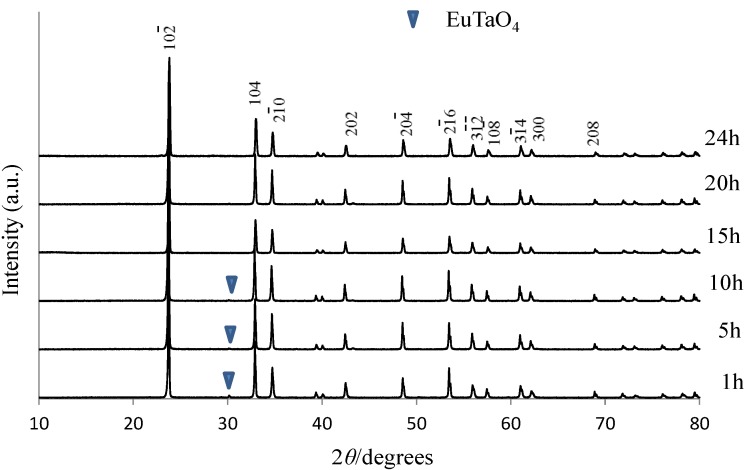
XRD patterns of Li_1.11_Ta_0.89_Ti_0.11_O_3_:Eu^3+^ phosphors for various annealing times.

In Eu^3+^-doped LNT phosphor, an annealing time of 24 h is preferable to that of 10 h because the superstructure forms during long-time annealing [10]. In the previous work, PL intensity might have been related to the structural distortion due to the superstructure. Indeed, PL intensity increased with increasing sintering time. In the LTT, the relationship between the annealing time and PL intensity should be clear. Figure 3 shows this relationship in Li_1.11_Ta_0.89_Ti_0.11_O_3_:Eu^3+^ phosphor. The results show that the best annealing time is 15 h. For shorter annealing time, a small amount of EuTaO_4_ was detected around 30 degrees in Figure 4. To diffuse the Eu^3+^ ion into the LiTaO_3_ structure homogeneously as an activator, the annealing time needs to be 15 h. We confirmed that the Eu^3+^ ion is randomly distributed over the Li site because the electron-density peak at the Li/Eu site of Eu-doped LTT was higher than that at the Li site of LTT [20]. The composition determined by the Rietveld method was (Li_0.977_Eu_0.023_)(Ta_0.89_Ti_0.11_)O_2.968_, which has a small amount of oxygen vacancies. The crystal structure of LiTaO_3_ is flexible with respect to the substitutions of Eu for Li and Ti for Ta, in which the <Li-O> distance of the (LiO_12_) polyhedron is 0.270 nm and 0.272 nm in Li(Ta_0.89_Ti_0.11_)O_2.945_ and (Li_0.977_Eu_0.023_)(Ta_0.89_Ti_0.11_)O_2.968_, respectively.

**Figure 5:**
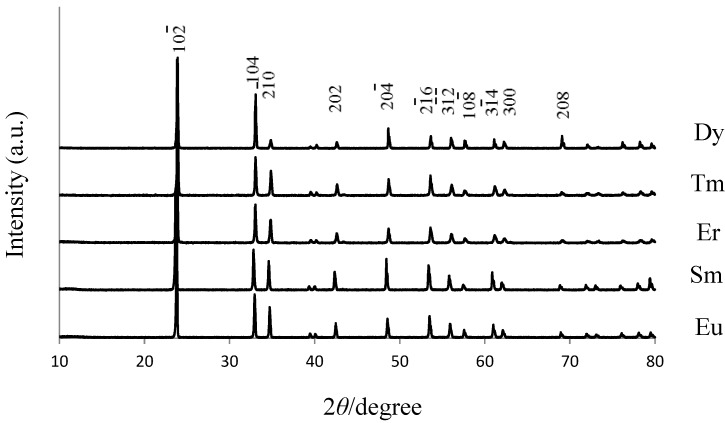
XRD patterns of Li_1.11_Ta_0.89_Ti_0.11_O_3_:RE^3+^ (RE: Dy. Tm, Er, Sm, Eu) for 15 h annealing.

**Figure 6:**
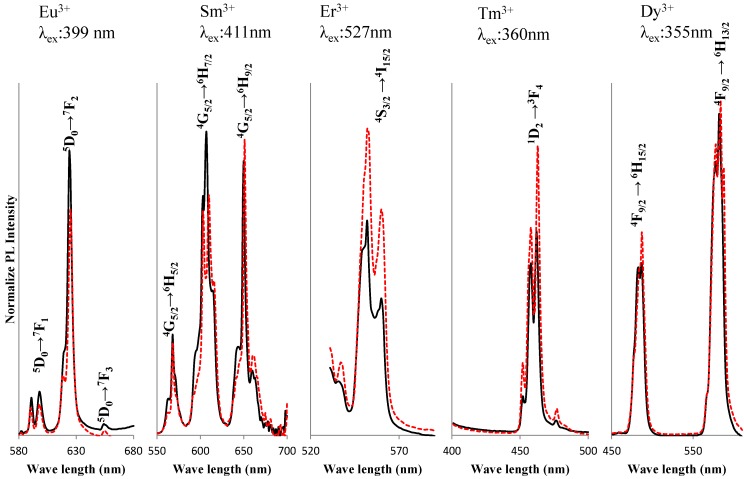
Comparison of PL intensity between LNT:RE^3+^ and LTT:RE^3+^. Dotted line: LNT:RE^3+^, solid line: LTT:RE^3+^.

The LTT phosphors with various emission colors were synthesized while doping with trivalent ions of Sm, Er, Tm and Dy. The host material of Li_1.11_Ta_0.89_Ti_0.11_O_3_ was used at a fixed RE_2_O_3_ concentration of 2.5 wt %. Figure 5 shows the XRD patterns of LTT:RE^3+^ phosphors annealed for 15 h. All structures were LiTaO_3_-type, and no impurity phase was detected. The results show that the rare-earth ion diffused into the host material homogeneously and that rare-earth ions might also be located at the Li site. Emission peaks were compared between LNT:RE^3+^ and LTT:RE^3+^ phosphor, as shown in Figure 6. It should be mentioned here that the respective f-f excitations were much stronger than any possible charge-transfer excitations for the present LNT:RE^3+^ and LTT:RE^3+^ phosphors. The excitation wavelengths were then chosen with the strongest f-f excitations observed in each phosphor. Red emission of the Eu^3+^-doped LTT was obviously brighter than that of LNT phosphor. Red emission was also observed for the Sm^3+^-doped LNT upon excitation at around 411 nm due to the ^6^H_5/2_-^6^P_3/2_ transition. Upon excitation at 411 nm, the photoluminescence spectra showed emission peaks at 568 nm (^4^G_5/2_-^6^H_5/2_), 607 nm (^4^G_5/2_-^6^H_7/2_), and 651 nm (^4^G_5/2_-^6^H_9/2_). The splitting of the 607 nm emission is prominent in LNT phosphor. Therefore it seems that the peak intensity ratio of LTT phosphor at 607/651 nm is larger than that of LNT phosphor. This would be caused by the difference in the overlapping of orbitals with the adjacent anions (Ta/Nb). A green emission peak at 527 nm (^4^S_3/2_-^4^I_15/2_) was observed for LNT:Er^3+^ upon excitation at 552 nm (^4^I_15/2_-^2^H_11/2_). Blue emission was observed at 360 nm (^1^D_2_-^3^F_4_ transition) for the LNT:Tm^3+^ phosphor upon excitation at 463 nm (^3^H_6_-^1^D_2_). Yellow emission peaks at 584 nm (^4^F_9/2_-^6^H_13/2_) were observed for LNT:Dy^3+^ upon excitation at 355 nm (^6^H_15/2_ -^4^M_15/2_). The highest emission peak intensity was compared between LNT:RE^3+^ and LTT:RE^3+^. Green and Blue emissions of LNT phosphor were brighter than those of LTT phosphor. The internal quantum efficiency of these phosphors were low level; 23% for LTT:Sm^3+^, 22% for LTT:Er^3+^, 35% for LTT:Tm^3+^, and 28% for LTT:Dy^3+^.

In contrast, the most effective activator was the Eu^3+^ ion in the LTT host material. The red emission intensity excited at 339 nm of LTT: Eu^3+^ phosphor is three times higher than that of Y_2_O_2_S: Eu^3+^ (4.3 at %), which is well known as a red phosphor. It is assumed that small differences in the lattice site between LNT and LTT host materials would affect the transition probability of the respective RE^3+^ ions. The combination of the host material and the emitting RE^3+^ ion is of great significance in designing new phosphors. A more detailed structural analysis, and the relevant theoretical calculations, will be performed in the near future.

Table 1 shows the chromaticity (*x*, *y*) of the LNT:RE^3+^ and LTT:RE^3+^ phosphors in the red, green, blue and yellow color regions. In general, the PL behavior of the RE^3+^-doped phosphors is not affected by the host material’s structure due to the 4f-4f transitions. In Sm phosphor, however, the difference in the emission intensity ratio at 606/650 nm affected the chromaticity.

**Table 1:** Chromaticity (*x*, *y*) of LNT and LTT phosphors.

Activator	LTT:RE^3+^		LNT:RE^3+^	
	*X*	*Y*	*X*	*Y*
Eu	0.673	0.327	0.676	0.324
Sm	0.622	0.378	0.630	0.370
Er	0.307	0.685	0.347	0.647
Tm	0.144	0.033	0.143	0.033
Dy	0.418	0.425	0.433	0.423

## 3. Experimental Procedure

The starting materials used were Li_2_CO_3_, Ta_2_O_5_ and TiO_2_ (>99.9% grade) to prepare the solid solution of LTT. The compositions of the LTT solid solutions prepared in this work followed the formula Li_1+*x*−*y*_Ta_1−*x*−3*y*_Ti*_x_*_+4*y*_O_3_ with 0.05 < *x* < 0.25, *y* = 0 as a host material. Rare earths (Eu_2_O_3_, Sm_2_O_3_, Er_2_O_3_, Tm_2_O_3_ and Dy_2_O_3_ > 99.9% grade) were doped in the host material. These powders were mixed and pressed in air at 1423 K for 1 h to 24h in a conventional electric furnace. As comparison materials, the rare earth doped Li_1+*x*_Nb_1−*x*_Ti*_x_*O_3_ (LNT) phosphors were also sintered at 1373 K for 24 h [13].

Structural analysis was carried out by X-ray diffraction (XRD) using a RINT 2500 (Rigaku Co., Ltd., Tokyo, Japan) operating at 40 kV and 200 mA. Microstructure images were observed by a scanning electron microscope (SEM) (SU8000, Hitachi Co., Ltd., Tokyo, Japan) operating at 3 kV. High-resolution TEM (HRTEM) images were observed by a device (2100 F, JEOL Co., Ltd., Tokyo, Japan) operating at 200 kV and equipped with energy-dispersed spectroscopy (EDS).

Excitation and emission spectra of the obtained samples were measured by a spectrometer (model FP-6500, JASCO international Co., Ltd., Tokyo, Japan). Quantum efficiency was measured by a spectral radiometer (MCPD-7000, Otsuka Electronics Co., Ltd., Osaka, Japan).

## 4. Conclusions

New phosphors have been successfully synthesized by doping rare-earth materials into a Li_1+*x*_Ta_1−*x*_Ti*_x_*O_3_ solid solution as a host material. The host material’s structure and photoluminescence (PL) property was compared to the RE^3+^-doped Li_1+*x*_Nb_1−*x*_Ti*_x_*O_3_ (LNT)_._ The resulting materials showed various color emissions, with LNT:Eu^3+^ and LNT:Sm^3+^ exhibiting red emission, LNT:Tm^3+^ blue emission, LNT:Er^3+^ green emission, and LTT:Dy^3+^ yellow emission. The host material’s structure of LTT phosphor with the optimal composition Li_1.11_Nb_0.89_Ti_0.11_O_3_ was not a superstructure. Therefore, the distortion due to superstructure did not affect the PL intensity in the LTT phosphor. The annealing time to synthesize the homogeneous phosphor was reduced from 24 h to 15 h. By doping Ti^4+^ and Eu^3+^ ions into the LiTaO_3_, the <Li-O> distance of the [LiO_12_] polyhedron in the LiTaO_3_ structure (*R*3*c*) changed without a superstructure. This change affected the interaction with the adjacent anions, and the emission from the Eu^3+^ ion in the Li site achieved a high value of internal quantum efficiency. In the LTT host material, the most effective activator was the Eu^3+^ ion. On the other hand, the emission intensities of Er- and Tm-doped LTT phosphors were lower that of LNT phosphor. We conclude that small differences in the lattice site between LNT and LTT host materials would affect the emission energy of the RE^3+^ ions.
